# Global model of low-frequency chorus (*f*_LHR_<*f*<0.1*f*_ce_) from multiple satellite observations

**DOI:** 10.1002/2013GL059050

**Published:** 2014-01-30

**Authors:** Nigel P Meredith, Richard B Horne, Wen Li, Richard M Thorne, Angélica Sicard-Piet

**Affiliations:** 1 British Antarctic Survey, Natural Environment Research CouncilCambridge, England; 2 Department of Atmospheric and Oceanic Sciences, University of CaliforniaLos Angeles, California, USA; 3 Office National d'Etudes et Recherches AérospatialesToulouse, France

**Keywords:** whistler mode chorus

## Abstract

Whistler mode chorus is an important magnetospheric emission, playing a dual role in the acceleration and loss of relativistic electrons in the Earth's outer radiation belt. Chorus is typically generated in the equatorial region in the frequency range 0.1–0.8 *f*_ce_, where *f*_ce_ is the local electron gyrofrequency. However, as the waves propagate to higher latitudes, significant wave power can occur at frequencies below 0.1*f*_ce_. Since this wave power is largely omitted in current radiation belt models, we construct a global model of low-frequency chorus, *f*_LHR_<*f*<0.1*f*_ce_, using data from six satellites. We find that low-frequency chorus is strongest, with an average intensity of 200 pT^2^, in the prenoon sector during active conditions at midlatitudes (20°<|*λ*_*m*_|<50°) from 4<*L*^∗^<8. Such midlatitude, low-frequency chorus wave power will contribute to the acceleration and loss of relativistic electrons and should be taken into account in radiation belt models.

**Key Points:**

1. Strong chorus waves can extend below 0.1 times local electron gyrofrequency
2. Low frequency chorus strongest at mid-latitudes in pre-noon sector for L*=4 to 8
3. Low frequency chorus should be included in radiation belt models

## 1. Introduction

Chorus is a discrete, naturally occurring whistler mode emission that is generated in two bands with a gap at 0.5 *f*_ce_ [*Tsurutani and Smith*, 1974], separating the emissions into so-called lower band (0.1*f*_ce_<*f*<0.5*f*_ce_) and upper band (0.5*f*_ce_<*f*<*f*_ce_) chorus. The waves are generated outside the plasmapause near the magnetic equator [*LeDocq et al.*, 1998] by cyclotron resonant interaction with suprathermal electrons [*Li et al.*, 2008] injected into the inner magnetosphere during storms and substorms. Consequently, chorus is found to be substorm dependent with the largest amplitudes being observed outside the plasmapause on the dawnside during enhanced geomagnetic activity [e.g., *Meredith et al.*, 2012], consistent with electron injection near midnight and subsequent drift through the dawnside.

Gyroresonant wave particle interactions with whistler mode chorus play a major role in radiation belt dynamics contributing to both the acceleration and loss of relativistic electrons [*Bortnik and Thorne*, 2007]. For example, chorus waves are thought to be largely responsible for the gradual buildup of radiation belt electrons that occurs on a time scale of 1–2 days during the recovery phase of geoeffective storms [e.g., *Horne et al.*, 2005]. In contrast, storm time chorus at mid to high latitudes causes microburst precipitation and may lead to losses of radiation belt electrons on the time scale of the order of a day [*Thorne et al.*, 2005]. There is also strong theoretical and observational evidence to suggest that chorus is the dominant source of plasmaspheric hiss [e.g., *Bortnik et al.*, 2008; *Meredith et al.*, 2013], which is itself responsible for the formation of the slot region between the inner and outer radiation belts [*Lyons and Thorne*, 1973] and the quiet time decay of energetic electrons in the outer radiation belt [*Meredith et al.*, 2006].

There are several dynamic global models of the radiation belts which are based on diffusion models [e.g., *Varotsou et al.*, 2005; *Fok et al.*, 2008; *Albert et al.*, 2009; *Subbotin et al.*, 2010; *Glauert et al.*, 2014]. They require diffusion rates that are proportional to the wave magnetic field intensity. *Horne et al.* [2013] recently computed energy and pitch angle diffusion rates for use in radiation belt codes using plasma wave observations of chorus in the frequency range 0.1–0.8 *f*_ce_ from seven satellites. Upper band chorus tends to be tightly confined to the magnetic equator [*Meredith et al.*, 2012]. On the other hand, lower band chorus can propagate to mid to high latitudes on the dayside [e.g., *Bunch et al.*, 2012; *Agapitov et al.*, 2013], where significant wave power can fall below 0.1 *f*_ce_ due to the increasing geomagnetic field strength. This power is largely omitted in current radiation belt models. To investigate the global distribution of this wave power and to improve the inputs to radiation belt models, here we develop a global model of chorus below 0.1 *f*_ce_, in the frequency range *f*_LHR_<*f*<0.1*f*_ce_, where *f*_LHR_is the lower hybrid resonance frequency, which we refer to as low-frequency chorus.

## 2. Instrumentation and Data Analysis

To construct a comprehensive database of low-frequency chorus in the inner magnetosphere, we combined data from six satellites. We used approximately 3 years of data from Dynamics Explorer 1, 10 years of data from Cluster 1, 1 year of data from Double Star TC-1, and 3 years of data from the Time History of Events and Macroscale Interactions during Substorms (THEMIS) A, D, and E satellites, respectively. The instrumentation, data analysis techniques, and the methods used to determine the location with respect to the plasmapause are described in *Meredith et al.* [2012]. The time resolution of the wave data used in this study, which ranges from 1 s to 32 s, is not sufficient to resolve individual chorus elements and identify chorus by its discrete nature. We therefore use the position of the waves with respect to the plasmapause to distinguish between chorus and plasmaspheric emissions, such as plasmaspheric hiss and lightning-generated whistlers, as done in recent studies to determine the global distribution of chorus from THEMIS [*Li et al.*, 2011] and multiple satellite [*Meredith et al.*, 2012] measurements. THEMIS wave data below 40 Hz suffers from a high level of noise, and so the THEMIS measurements were flagged whenever *f*_LHR_ fell below 40 Hz and excluded from the subsequent analysis. We then binned the low-frequency chorus (*f*_LHR_<*f*<0.1*f*_ce_) from each satellite as a function of *L*^∗^, magnetic local time (MLT), magnetic latitude (*λ*_*m*_), and geomagnetic activity as monitored by the *AE* index as detailed in Table 1. Here *L*^∗^ is related to the third adiabatic invariant and may be thought of as the radial distance in Earth radii to the equatorial locus of the symmetric shell on which particles would be found if the nondipolar components of the trapping field were adiabatically removed [*Roederer*, 1970]. For the database, *L*^∗^ and MLT were computed using the Office National d'Etudes et de Recherches Aérospatiale Département Environnement Spatiale (ONERA-DESP) library V4.2, [*Boscher et al.*, 2008] with the International Geomagnetic Reference Field at the middle of the appropriate year and the Olson-Pfitzer quiet time model [*Olson and Pfitzer*, 1977]. Since the software is designed for particles and we are using it for waves, we assume a local pitch angle of 90° in the calculation of *L*^∗^. We then combined the data from each of the satellites, weighting the data from each individual satellite by the corresponding number of samples, to produce a combined wave database as a function of *L*^∗^, MLT, *λ*_*m*_, and geomagnetic activity. For the resulting maps presented below, we excluded measurements from any given spatial bin when the number of samples was less than 5.

**Table 1:** Format of the Low-Frequency Chorus Database

Parameter	Bins
*L*^∗^	90 linear steps from *L*^∗^ = 1 to *L*^∗^ = 10
MLT	24 linear steps from 0 MLT to 24 MLT
*λ*_*m*_	60 linear steps from −90° to 90°
Activity	10 activity levels as monitored by *AE*

## 3. Results

**Figure 1:**
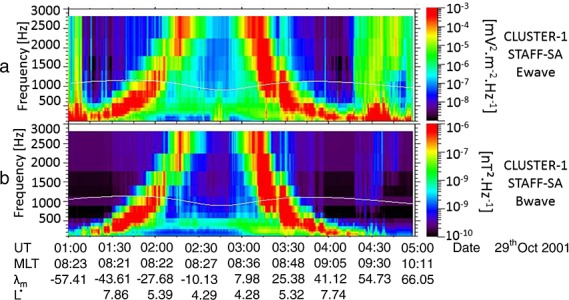
Cluster 1 measurements of (a) the total spin plane wave electric power spectral density and (b) the total wave magnetic power spectral density as a function of frequency and UT time from 01:00 to 05:00 UT on 29 October 2001. The solid line in both panels represents 0.1 *f*_ce_.

Figure 1 shows an example of low-frequency chorus observed on Cluster 1 during a perigee pass that occurred during the recovery phase of the 28 October 2001 geomagnetic storm. Figure 1a shows the total wave electric power spectral density in the spin plane from the Electric Fields and Wave experiment [*Gustafsson et al.*, 1997], and Figure 1b shows the total wave magnetic power spectral density from the Spatio-Temporal Analysis of Field Fluctuations experiment [*Cornilleau-Wehrlin et al.*, 1997] as a function of frequency and UT time from 01:00 to 05:00 UT on 29 October 2001. The magnetic local time, magnetic latitude, and the value of *L*^∗^ are also given at 30 min intervals. The solid white line in Figures 1a and 1b represents 0.1 *f*_ce_, determined from the fluxgate magnetometer on board Cluster 1. Strong chorus waves are observed during this interval, as evidenced by the enhanced power spectral density in both the electric and magnetic wave field components, from 01:00 UT to 04:00 UT. Strong low-frequency chorus is seen at absolute magnetic latitudes greater than ∼25°, both north and south of the magnetic equator, with little or no power above 0.1 *f*_ce_ at these latitudes. Closer to the equator the chorus wave power is largely above 0.1 *f*_ce_, with little or no low-frequency chorus, illustrating that chorus wave power tends to fall below 0.1 *f*_ce_ at midlatitudes.

### 3.1. Global Morphology of Low-Frequency Chorus

#### 3.1.1. MLT Distribution

**Figure 2:**
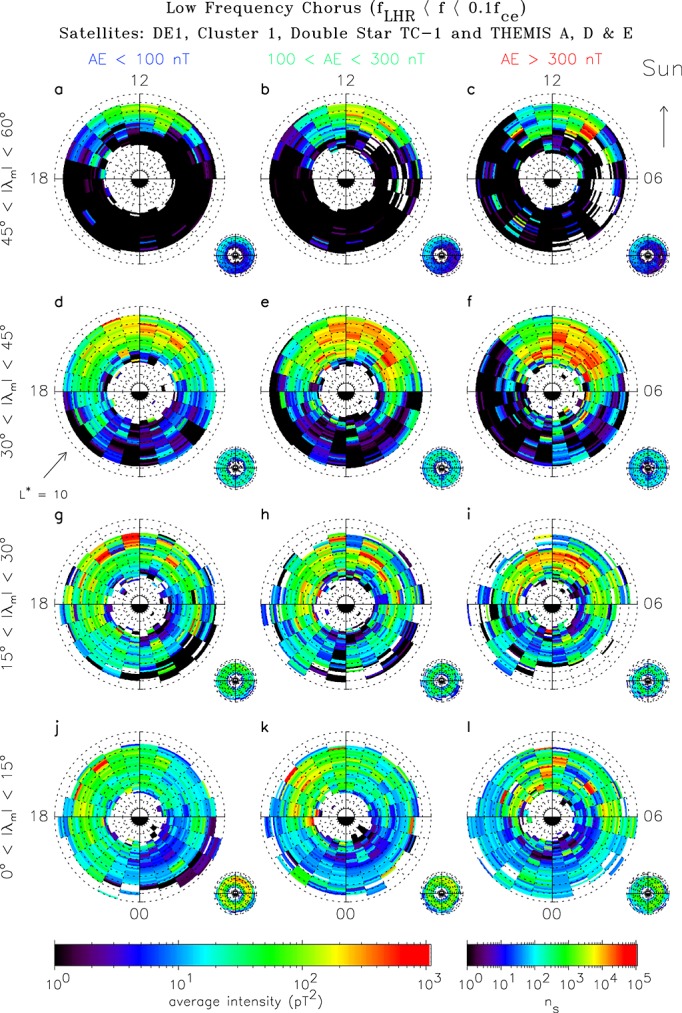
Global maps of the average wave intensity of low-frequency chorus as a function of *L*^∗^ and MLT for, from bottom to top, increasing magnetic latitude and, from left to right, increasing geomagnetic activity. The plots extend linearly out to *L*^∗^ = 10 with noon at the top and dawn to the right. The average intensities are shown in the large panels and the corresponding sampling distributions in the small panels.

The global distribution of low-frequency chorus is shown as a function of geomagnetic activity, |*λ*_*m*_|, *L*^∗^, and MLT in Figure 2. Each plot extends linearly out to *L*^∗^ = 10 with noon at the top and dawn to the right. The average intensities are shown in the large panels and the corresponding sampling distributions in the small panels. The data have been averaged over both hemispheres into bins of width 0.2 *L*^∗^.

Low-frequency chorus tends to be rather weak in the equatorial region, 0° <|*λ*_*m*_|< 15° (Figures 2j–2l), with typical intensities of the order of tens of pT^2^. In contrast, at midlatitudes, 15° <|*λ*_*m*_|< 30° (Figures 2g–2i) significant wave power is observed, but it is restricted to the dayside. Here the waves are substorm dependent with the largest intensities being seen during active conditions with intensities of the order several hundred pT^2^ from 4 <*L*^∗^< 7 in the region 08 <*MLT*< 15. At higher midlatitudes, 30° <|*λ*_*m*_|< 45° (Figures 2d–2f), the low-frequency chorus is again strongest and most extensive on the dayside during active conditions, with intensities of the order several hundred pT^2^ from 4 <*L*^∗^< 8 in the region 07 <*MLT*< 15. Interestingly, moderate wave power is also observed during quiet conditions at large *L*^∗^ in the noon (09–15 MLT) sector. The low-frequency chorus wave power drops off in both magnitude and extent at high latitudes 45° <|*λ*_*m*_|< 60° (Figures 2a–2c), where weak power of the order of tens of pT^2^ is restricted to the noon sector beyond *L*^∗^ = 7. Low-frequency chorus tends to be largely absent on the nightside at all magnetic latitudes and for all levels of geomagnetic activity.

#### 3.1.2. Latitudinal Distribution

**Figure 3:**
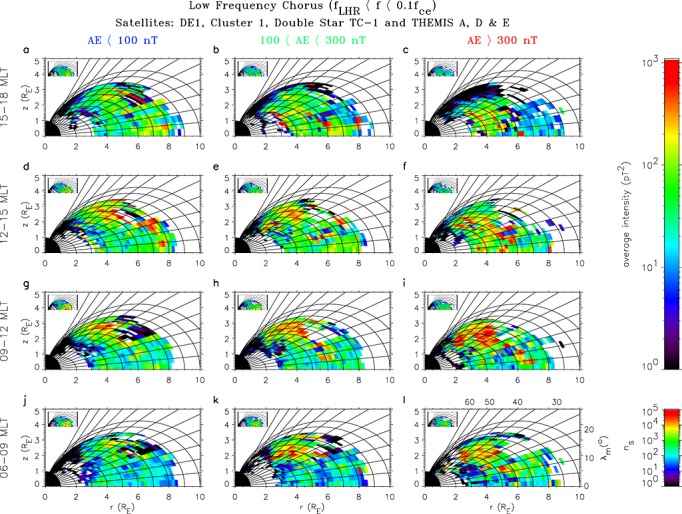
Global maps of the average wave intensity of low-frequency chorus in the meridional plane on the dayside for, from bottom to top, increasing MLT and, from left to right, increasing geomagnetic activity. The average intensities are shown in the large panels and the corresponding sampling distributions in the small panels.

The global distribution of low-frequency chorus is shown as a function of geomagnetic activity and magnetic local time in the meridional plane on the dayside in Figure 3. The data have again been averaged over both hemispheres into bins of width 0.2 *L*^∗^. Dipole field lines and lines of constant magnetic latitude are included to help visualise the behavior of the wave intensities as a function of *L*^∗^ and |*λ*_*m*_|. The average intensities are shown in the large panels and the corresponding sampling distributions in the small panels. The strong waves on the dayside during active conditions are most intense in the prenoon sector and extend from 20° to 50° (Figure 3i), with an average intensity of 200 pT^2^ in this region for 4 <*L*^∗^< 8. In contrast, there is very little power in the equatorial region below about 20°, confirming that these waves are largely a midlatitude phenomenon.

## 4. Discussion

Lower band chorus waves generated near the magnetic equator in the frequency range 0.1 *f*_ce_–0.5*f*_ce_ fall to lower relative frequencies as they propagate to higher latitudes and hence may fall into the low-frequency chorus band as defined here. Low-frequency chorus is not observed on the nightside because waves at higher relative frequencies generated near the equator in the lower band are confined to latitudes less than 15° [*Meredith et al.*, 2012] due to strong Landau damping by suprathermal electrons [*Bortnik et al.*, 2007]. On the dayside, however, the flux of suprathermal electrons is much lower and chorus generated near the equator in the lower band propagates to higher latitudes and lower relative frequencies where it may be observed as low-frequency chorus. For example, lower band chorus (0.1*f*_ce_<*f*<0.5*f*_ce_) is typically observed up to about 30° on the dayside [*Meredith et al.*, 2012] but is largely absent above this latitude. At lower frequencies, in the range *f*_LHR_<*f*<0.1*f*_ce_, there is little wave power, on average, near the magnetic equator but significant wave power, of the order several hundred pT^2^ can be observed at midlatitudes, from 20° to 50°, in the region 4<*L*^∗^< 8 from 07 to 15 MLT.

*Bunch et al.* [2012] recently conducted a study of off-equatorial chorus wave intensities using data from the Polar Plasma Wave Investigation. However, due to its high inclination orbit, the latitude coverage depends strongly on *R*_*o*_, the radial distance to the equatorial field line crossing, with coverage ranging from approximately 15°–35° at *R*_*o*_ = 5–6 to approximately 25°–40° at *R*_*o*_ = 7–8 near dawn. In contrast, our database covers most of the inner magnetosphere enabling us to observe and quantify the full spatial extent of the waves, as required by radiation belt models. Furthermore, we determine the wave power directly by integrating the power spectral density over the appropriate frequency band defined using the local electron gyrofrequency, whereas *Bunch et al.* [2012] estimate the wave power from the measured chorus peak spectral intensity assuming a Gaussian profile with a fixed peak position and width at the minimum field line gyrofrequency. In addition to assuming a spectral shape this technique also assumes that the waves have traveled to the satellite along the magnetic field. However, chorus at mid to high latitudes may be highly oblique [e.g., *Agapitov et al.*, 2013]. Such waves cannot be simply mapped back to the equator along the local geomagnetic field as they can propagate across the field. Indeed, ray tracing studies show that these waves may refract inward or outward from the magnetic field direction as they propagate to higher latitudes [e.g., *Bortnik et al.*, 2008], by as much as 1 *L*^∗^ or more, which could result in incorrect mapping of the wave power in both position and relative frequency. It is difficult to make a precise numerical comparison with the statistical results in *Bunch et al.* [2012] due to differences in satellite coverage in the various MLT sectors. For a “rough” comparison, we note that *Bunch et al.* [2012] calculate the time-averaged amplitudes for high-latitude chorus (15°–45°, 4–8 R_E_) for *AE*> 100 nT to be 1.0, 6.7, 18, and 0.06 pT in the midnight, dawn noon, and dusk sectors, respectively. Our averaged chorus amplitudes for the same regions and activity conditions, which include contributions from low-frequency and lower band chorus, are 3.6, 11 and 14.5, and 7.5 pT respectively. The large-scale average results compare favorably near dawn and noon. However, there is a very large difference near dusk, but this is most likely due to the very limited coverage of the Polar spacecraft in this sector.

Several radiation belt models currently use models of upper and lower band chorus defined using the local electron gyrofrequency [*Varotsou et al.*, 2005; *Fok et al.*, 2008; *Albert et al.*, 2009; *Glauert et al.*, 2014] and do not include wave power below 0.1 *f*_ce_. Our results show that significant wave power extends below 0.1 *f*_ce_ particularly at midlatitudes in the prenoon sector where it may have a significant effect on radiation belt dynamics. *Thorne et al.* [2005] showed that significant scattering near the loss cone can occur for relativistic electrons but only at latitudes greater than 30°. This suggests that midlatitude chorus is required to produce million electron volts microbursts. Our results show that midlatitude chorus is largely restricted to the prenoon sector consistent with the region of maximum microburst precipitation [e.g., *O'Brien et al.*, 2003]. For medium to high energy electrons, midlatitude chorus at absolute magnetic latitudes greater than 20° is likely to extend the range of pitch angles over which acceleration is possible [e.g., *Bunch et al.*, 2013] and extend the potential range of energization to higher energies, but only at intermediate pitch angles. The model of the wave power presented here can be used, together with appropriate models of the plasma density [*Horne et al.*, 2013] and wave normal angle distribution [*Agapitov et al.*, 2013], to compute the associated diffusion rates for use in global radiation belt models [e.g., *Glauert et al.*, 2014].

## 5. Conclusions

We have conducted a global statistical survey of low-frequency chorus (*f*_LHR_<*f*<0.1*f*_ce_) using plasma wave data from six satellites. Our main conclusions are

1. Low-frequency chorus is substorm dependent with strongest intensities during active conditions.
2. The waves are strongest on the dayside with an average intensity of 200 pT^2^ in the prenoon sector during active conditions at midlatitudes (20°<|*λ*_*m*_|<50°) from 4<*L*^∗^<8.
3. Low-frequency chorus tends to be largely absent on the nightside at all magnetic latitudes and for all levels of geomagnetic activity.

Significant low-frequency chorus wave power is observed during active conditions over a wide region of geospace on the dayside at midlatitudes. Such waves will contribute to the acceleration and loss of relativistic electrons and should be included in radiation belt models.
